# Screening for late-onset Pompe disease in Internal Medicine departments in Spain

**DOI:** 10.1186/s13023-023-02887-z

**Published:** 2023-08-31

**Authors:** Mónica López-Rodríguez, Miguel Angel Torralba-Cabeza, Iván Pérez de Pedro, Alberto Rivera, Roi Suarez Gil, Ana Gómez-Belda, Jose Luis Patier de la Peña, Alberto de los Santos Moreno, Albert Selva-O’Callaghan, Igor Gómez Gárate, Andrés González García, Roberto Hurtado, Pablo Tutor de Ureta, Miguel Ángel Barba-Romero, José C. Milisenda, Josep M. Grau-Junyent, POMPE study group

**Affiliations:** 1 Internal Medicine Department, Ramón y Cajal University Hospital, Madrid, Spain; 2 Internal Medicine Department, Lozano Blesa University Hospital, Zaragoza, Spain; 3 Internal Medicine Department, Málaga Regional University Hospital, Málaga, Spain; 4 Internal Medicine Department, University Hospital Complex of Vigo, Vigo, Spain; 5 Internal Medicine Department, Lucus Augusti University Hospital, Lugo, Spain; 6 Internal Medicine Department, Dr. Peset University Hospital, Valencia, Spain; 7 Internal Medicine Department, Puerta del Mar University Hospital, Cádiz, Spain; 8 Internal Medicine Department, Vall d’Hebron General Hospital, Barcelona, Spain; 9 Internal Medicine Department, Araba University Hospital, Álaba, Spain; 10 Internal Medicine Department, Vega Baja Hospital, Alicante, Spain; 11 Internal Medicine Department, Puerta de Hierro-Majadahonda University Hospital, Madrid, Spain; 12 Internal Medicine Department, Albacete University Hospital, Albacete, Spain; 13 Internal Medicine Department, Hospital Clínic, University of Barcelona and CIBERER (Madrid), C/Villarroel 170, 08036 Barcelona, Spain

**Keywords:** Late-onset pompe disease, Internal medicine department, Screening, Spain, Dried blood spots

## Abstract

**Background:**

The screening of high-risk populations using dried blood spots (DBS) has allowed the rapid identification of patients with Pompe disease, mostly in Neurology departments. The aim of the study was to determine the prevalence of late-onset Pompe disease (LOPD) among patients not previously diagnosed or tested for this entity despite presenting possible signs or symptoms of the disease in Internal Medicine departments in Spain.

**Methods:**

This epidemiological, observational, cross-sectional, multicenter study included a single cohort of individuals with clinical suspicion of LOPD seen at Internal Medicine departments in Spain. The diagnosis of LOPD was initially established on the basis of the result of DBS. If decreased enzyme acid-alpha-1,4-glucosidase (GAA) activity was detected in DBS, additional confirmatory diagnostic measurements were conducted, including GAA activity in lymphocytes, fibroblasts, or muscle and/or genetic testing.

**Results:**

The diagnosis of LOPD was confirmed in 2 out of 322 patients (0.6%). Reasons for suspecting LOPD diagnosis were polymyositis or any type of myopathy of unknown etiology (in one patient), and asymptomatic or pauci-symptomatic hyperCKemia (in the other). The time between symptom onset and LOPD diagnosis was 2.0 and 0.0 years. Both patients were asymptomatic, with no muscle weakness. Additionally, 19.7% of the non-LOPD cases received an alternative diagnosis.

**Conclusions:**

Our study highlights the existence of a hidden population of LOPD patients in Internal Medicine departments who might benefit from early diagnosis and early initiation of potential treatments.

**Supplementary Information:**

The online version contains supplementary material available at 10.1186/s13023-023-02887-z.

## Background

Pompe disease (PD), also known as glycogen storage disease type II, is a rare, autosomal recessive disorder of the metabolism characterized by the deficiency of the lysosomal enzyme acid-alpha-1,4-glucosidase (GAA) (EC 3.2.1.20) [1]. It leads to the progressive accumulation of intra- and extralysosomal glycogen, especially in skeletal and cardiac muscles [2]. The *GAA* gene is localized on the long arm of chromosome 17 [3]. More than 350 variants have been identified so far, the c.-32-13 T > G being the most frequent variant in Caucasian PD patients [4]. Two forms of PD have been distinguished, i.e. infantile-onset PD (IOPD) and late-onset PD (LOPD). IOPD is the most severe presentation and is characterized by the age of onset (≤ 12 months), hypertrophic cardiomyopathy, hypotonia, muscle weakness, and respiratory failure [1, 5]. The LOPD form, known as the juvenile or adult form of PD (because symptoms onset occurs at an age over 12 months), shows a less devastating but more heterogeneous clinical phenotype. LOPD may vary from asymptomatic elevated creatine kinase (CK) activity in serum (hyperCKemia) to weakness and atrophy of proximal, axial, and paravertebral muscles as well as respiratory failure [6].

Early detection of PD and early initiation of treatment have been associated with improved ambulation and survival, as well as prevention of respiratory function deterioration [7]. Currently, enzyme replacement therapy is the only available treatment [8]. Yet, the clinical variability of LOPD and the overlap of signs and symptoms with other neuromuscular disorders (for instance, limb girdle muscular dystrophies, Duchenne muscular dystrophy, or polymyositis) frequently leads to a considerable delay in diagnosis and treatment [9, 10]. In 2017, the European Pompe Consortium, among other recommendations on diagnosis and management, proposed the measurement of GAA activity in dried blood spots (DBS) as an appropriate first-line diagnostic test for PD, followed by a further confirmatory diagnostic measurement, such as GAA activity in leukocytes, fibroblasts, or skeletal muscle and/or demonstration of variants in the *GAA* gene [11]. The screening of high-risk populations using DBS has allowed the rapid identification of patients [9, 12–16]; however, most patients were seen at Neurology departments.

Therefore, the aim of this study was to determine the prevalence of LOPD among patients not previously diagnosed or tested for this entity despite presenting possible signs or symptoms of the disease, evaluated in Internal Medicine departments in Spain and considering the phenotype involved (symptomatic or asymptomatic).

## Methods

### Study design

This epidemiological, observational, cross-sectional, multicenter study included a single cohort of individuals with clinical suspicion of LOPD seen at Internal Medicine departments in Spain. A total of 13 hospitals participated in the study. The study included adult patients (≥ 18 years) who presented with at least one of the following clinical criteria: polymyositis or any type of myopathy of unknown etiology; diagnosis of obstructive sleep apnea syndrome (OSAS) by polysomnography together with a body mass index (BMI) ≤ 30 kg/m^2^; asymptomatic or pauci-symptomatic hyperCKemia, according to Kyriakides recommendations [17]; or asymptomatic or pauci-symptomatic hyperCKemia (CK > 1.5 times upper limit of normal, ULN) in patients who are on statins. Participating investigators identified patients who met the inclusion criteria in their consultations and invited them to participate in the study during a routine follow-up visit. None of the invited patients declined such participation. At this time, the patients signed an informed consent form, and a blood sample was obtained for the dried blood sample test. Patients with clinical suspicion of LOPD were then prospectively included in the study. The patient recruitment period lasted from June 2018 to September 2022. The study was conducted in accordance with the Declaration of Helsinki, and approved by an independent Ethics Committee.

### Diagnosis of LOPD

The diagnosis of LOPD was initially established based on the result of the DBS. The study of total (neutral and acid) GAA activity in DBS was determined using 4-methylumbelliferyl-alpha-D-glucoside as the substrate. A significantly diminished GAA activity in DBS was considered when it showed a value lower than the normal range (0.8–5.0 μmol/L/h). The enzyme activity inhibited by acarbose at acid pH was also determined. Acarbose specifically inhibits non-lysosomal maltase and allows calculation of the effective GAA by the quotient of total neutral GAA and the enzyme activity inhibited by acarbose. A percentage of GAA inhibition higher than 80% was considered pathological (calculated as total GAA activity-GAA activity inhibited by acarbose/total GAA activity). If decreased GAA activity in DBS was detected, additional confirmatory diagnostic measurements were carried out, including GAA activity in lymphocytes, fibroblasts, or muscle and/or genetic testing. Tetrasaccharide glucose study in urine or blood smear to evaluate vacuolated PAS-positive lymphocytes was not carried out in any case because the physician in charge of each patient always found an alternative etiologic that justified the elevation of CK. All procedures were performed in a single reference center (University Hospital Virgen del Rocio, Seville, Spain).

In several centers, after LOPD was ruled out (i.e. non-PD patients), subjects were followed up in order to explore their alternative diagnosis. In such cases, the final diagnosis was carried out by the combination of muscle biopsy, electromyogram, ischemic forearm test, or genetic tests.

### Descriptive analyses

Continuous variables were quantified as means and the standard deviations (SD) or medians and interquartile ranges (IQR), as appropriate. Categorical variables were calculated as counts and percentages. Missing data were not considered in the analyses. All analyses were performed with the SAS Enterprise Guide version 8.3.

## Results

### Study population

**Fig. 1:**
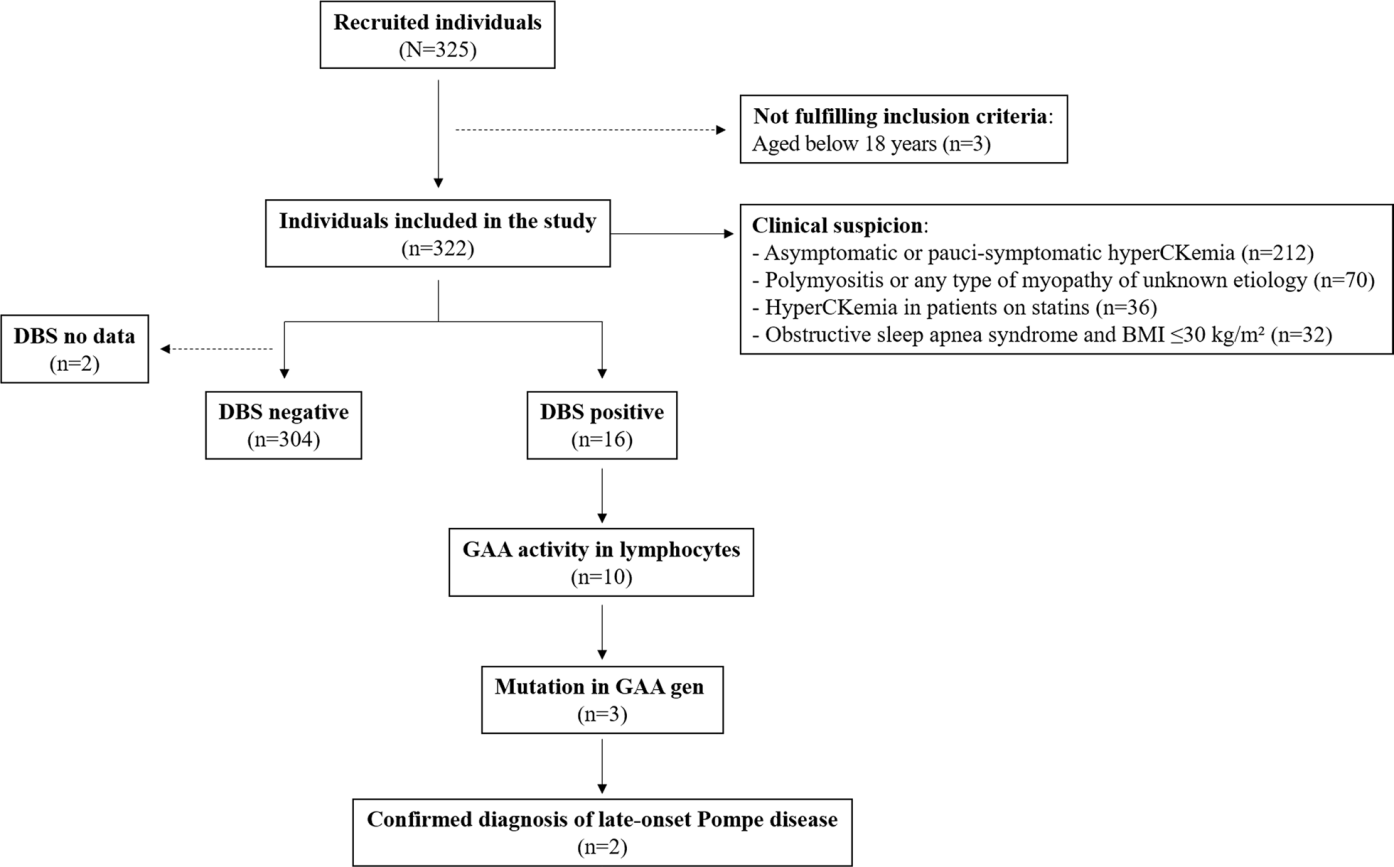
Overall patient flowchart. BMI, body mass index; DBS, dried blood spots; GAA, acid-alpha-1,4-glucosidase

At the end of the study, 325 subjects were enrolled in the database. However, three were excluded for not fulfilling the inclusion criteria (aged below 18 years). A total of 322 individuals were finally included (Fig. 1). The distribution of included patients by hospital and Autonomous Community in Spain are shown in the Additional file 1: Table S1, Fig. S1. Patients were predominantly male (63.7%), with a median age of 47 years (IQR, 35–58), and a median BMI of 26.2 kg/cm^2^ (IQR, 23.5–29.1). Sociodemographic and clinical characteristics of individuals screened for PD and included in the study are shown in Table 1. Most subjects presented with asymptomatic or pauci-symptomatic hyperCKemia (65.8% of total), followed by polymyositis or any type of myopathy of unknown etiology (21.7%). GAA activity in DBS was decreased in 16 patients (5.0%). Three patients (0.9%) showed variants in the *GAA* gene.

**Table 1:** Sociodemographic and clinical characteristics of screened individuals

Individuals' characteristics	Number of available patients	Value
Age, median (IQR), years	322	47 (35–58)
Gender, male, n (%)	322	205 (63.7)
BMI at study inclusion, median (IQR), kg/cm^2^	322	26.2 (23.5–29.1)
Clinical suspicion, n (%)		
Asymptomatic or pauci-symptomatic hyperCKemia	322	212 (65.8)
Polymyositis or any type of myopathy of unknown etiology	322	70 (21.7)
HyperCKemia in patients on statins	322	36 (11.2)
Obstructive sleep apnea syndrome and BMI ≤ 30 kg/m^2^	322	32 (9.9)
Decreased GAA activity	320	16 (5.0)
Tissue used for determining the decreased GAA activity ^*^	16	
In DBS, n (%)	10	10 (62.5)
Percentage of GAA inhibition in DBS, median (IQR)	10	81.5 (78.0–84.0)
In lymphocytes, n (%)	6	6 (37.5)
Percentage of GAA inhibition in lymphocytes, median (IQR) %	6	73.5 (68.0–82.0)
In muscle, n (%)	1	1 (6.3)
Percentage of GAA inhibition in muscle, %	1	50.0
Number of patients with serum CK lower than ULN, n (%) ^**^	10	3 (30.0)
Number of patients with serum CK higher than ULN, n (%) ^**^	10	7 (70.0)
Genetic study, n (%)		
No GAA variants	5	2 (40.0)
GAA variant	5	3 (60.0)
c.-32-13 T > G	3	2 (66.7)
c.1443G > A	3	1 (33.3)
c.854C > G	3	1 (33.3)

*Multiresponse, i.e. one patient could have two samples for confirming decreased GAA activity

**Calculated over the 16 patients with decreased GAA activity

*IQR* Interquartile range; *BMI* Body mass index; *GAA* Acid-alpha-1,4-glucosidase; *ULN* Upper limit of normal

### Confirmation of LOPD diagnosis

The diagnosis of LOPD was confirmed in two patients (0.6% of all investigated) only. An additional genetic study performed in one of the three patients harbouring a variant in *GAA* gene revealed that it was actually a non-LOPD case. Sociodemographic and clinical characteristics of patients with LOPD are shown in Table 2. Both were male and Caucasian. Their age at symptom onset was 16 and 51 years, respectively. The time between symptom onset and LOPD diagnosis was 2.0 and 0.0 years. The clinical suspicion for study inclusion was polymyositis or any type of myopathy of unknown etiology (in one patient), and asymptomatic or pauci-symptomatic hyperCKemia (in the other one). Both were asymptomatic, with neither muscle weakness nor respiratory insufficiency. The PD patients showed the variants c.-32-13 T > G, and one also had c.1443G > A. Thus, one patient was homozygous for c.-32-13 T > G and the other was compound heterozygous.

**Table 2:** Sociodemographic and clinical characteristics of patients with confirmed LOPD (n = 2)

	Patient 1	Patient 2
Gender	Male	Male
Race	Caucasian	Caucasian
Age at symptom onset, years	16	51
Age at diagnosis, years	18	51
Time between symptom onset and Pompe diagnosis, years	2	0
Cause of study inclusion	Polymyositis or any type of myopathy of unknown etiology	Asymptomatic or pauci-symptomatic hyperCKemia
Phenotype of the disease	Asymptomatic	Asymptomatic
Weakness	No weakness	No weakness
Exercise intolerance	No	No
Respiratory insufficiency	No	No
Magnetic resonance imaging		
Shoulder girdle	Normal	Normal
Pelvis	Normal	Normal
Lower extremities	Normal	Abnormal
Upper extremities	Normal	Normal
Distal muscles	Normal	Normal
Axial muscles	Normal	Abnormal
Percentage of GAA inhibition		
In DBS	93.0	50.0
In lymphocytes	82.0	Not performed
In muscle	Not performed	Not performed
Maximum serum CK, IU/L	1,107	201
Maximum serum CK, IU/L	417	201
Laboratory ULN	192	300
GAA variants	c.-32-13 T > G and c.1443G > A	c.-32-13 T > G (homozygous)
Receiving treatment with ERT	No	No

*CK* Creatine kinase; *BMI* Body mass index; *DBS* Dried blood spots; *GAA* Acid-alpha-1,4-glucosidase; *ULN* Upper limit of normal; *IU* International unit; *ERT* Enzyme replacement therapy

### Diagnosis of non-LOPD patients

Once PD was discarded, the establishment of a final diagnosis was followed up in 213 cases. Of these, an alternative diagnosis (other than PD) was obtained for 42, thus representing 19.7% of the series (Table 3). Most diagnoses were metabolic disorders (n = 12), myositis (n = 12), and muscle dystrophies (n = 10).

**Table 3:** Diagnosis of non-LOPD patients

	n
Metabolic disorders	12
McArdle disease	7
Carnitine palmitoyltransferase II deficiency	3
Acyl-CoA dehydrogenase multiple deficiency	2
Myositis	12
Immune-mediated necrotizing myopathy (statin-related)	8
Inclusion body myositis	3
Dermatomyositis	1
Muscle dystrophies	10
Anoctamin 5	3
LMNA-related muscle dystrophies	1
Duchenne carrier	1
Distrobrevin	1
Nonaka myopathy	1
Limb-girdle muscular dystrophy type 1G	1
Facioscapulohumeral muscular dystrophy	1
Becker muscular dystrophy	1
Other single conditions	8
Amyotrophic lateral sclerosis	1
McLeod syndrome	1
Lambert-Eaton myasthenic syndrome	1
Bethlem myopathy	1
Pseudoxantoma elasticum	1
Multicore myopathy	1
Hereditary neuropathy	1
Mitochondrial myopathy	1

## Discussion

The present study adds further evidence to the very low prevalence of LOPD (0.6%) from a cohort of individuals who were seen at the Internal Medicine departments of 13 hospitals in Spain and presented no conclusive diagnosis, mainly myopathies of unknown etiology. The prevalence of LOPD is similar, although slightly lower than that reported in previous studies worldwide [6, 7, 9, 13–16, 18–24] and in Spain [10, 12]. For instance, Preisler et al. screened for PD in two neuromuscular clinics and one respiratory center from Denmark, involving 103 subjects [13]. Three patients (2.9%), with unclassified limb-girdle muscular dystrophy, had confirmed diagnosis of PD. Spada et al. [15] evaluated PD diagnosis in 137 patients with unexplained hyperCKemia in Neuroscience and Pediatrics departments in Italy. Three patients (2.2%) were diagnosed with LOPD. Lukacs et al. screened 3076 patients with hyperCKemia and/or limb-girdle muscular weakness from seven German and British neuromuscular centers [14].

A total of 76 patients (2.4%) received the diagnosis of PD. Golsary et al. [6] evaluated the prevalence of LOPD in 69 patients with limb-girdle muscle weakness and/or hyperCKemia and undiagnosed muscle biopsy who underwent a DBS from a German neuromuscular center. The diagnosis of LOPD was established in two of the subjects (2.9%). Regarding studies in Spain, Pérez-López et al. [10] evaluated the prevalence of LOPD in 140 patients with a myopathy of unknown etiology or idiopathic rise of CK levels from an Internal Medicine department. Two patients (1.4%) were finally diagnosed with LOPD. In addition, Gutierrez-Rivas et al. [12] in a prospective, multicenter, observational study involving 146 patients with unclassified limb-girdle muscular dystrophy and 202 with asymptomatic or pauci-symptomatic hyperCKemia, confirmed the LOPD diagnosis in 16 (4.6%).

In our study, the slightly lower prevalence of LOPD (0.6%) compared with previous studies might derive from including individuals only from Internal Medicine departments, instead of from other departments more specialized in neuromuscular disorders. Indeed, the prevalence in our study is closer to the prevalence observed in the study by Pérez et al. [10] (1.4%), which also included patients from an Internal Medicine department. Furthermore, our patients with confirmed LOPD had clinical suspicion of polymyositis or any type of myopathy of unknown etiology (one patient) or asymptomatic or pauci-symptomatic hyperCKemia (the other one). These conditions (polymyositis or asymptomatic hyperCKemia) are part of the differential diagnosis of LOPD, together with a number of additional diseases [4]. In general, according to diagnostic and management guidelines, symptoms such as muscle fatigue, clumsiness, difficulty in breathing, and/or elevated muscle enzymes may be suggestive of LOPD diagnosis [4].

The difficulty in diagnosis usually results in a diagnostic delay, averaging approximately seven years [9, 10, 12–14, 25]. Reducing diagnostic delay is important because it provides the possibility to initiate the only current treatment available (enzyme replacement therapy) earlier [8]. In our study, the diagnostic delay was markedly low (ranging from zero to two years), compared to other reports [6, 7, 9, 12, 13, 16, 22, 24]. A prior study at an Internal Medicine department in Spain reported the case of one patient that spent eight years from the onset of his symptoms to final diagnosis of LOPD [25]. If the evaluation of these patients seen in the Internal Medicine departments had not been performed, these patients with LOPD could have remained with no conclusive diagnosis for a longer time. Clinical suspicion of PD is extremely important for medical professionals, regardless of their specialty, in subjects presenting with the previously described symptoms in order to smooth the patient journey as much as possible.

On the other hand, as PD progresses, respiratory impairments may appear during sleep, including OSAS [4]. Thus, OSAS could also be indicative of LOPD diagnosis. Despite this, none of our patients with OSAS was finally diagnosed with LOPD. Similarly, Stolk et al. [26] evaluated the diagnosis of LOPD in a study involving 544 patients with mild to severe OSAS, and did not find any confirmed case of LOPD.

One limitation of our study might be related to the multicenter design since department policies may be quite different depending on the regional healthcare recommendations. On the other hand, 42 patients received a final diagnosis of non-PD, 15 with a treatable condition, and genetic counseling in the remaining cases. This shows that it is possible to reach a definitive diagnosis of this type of rare diseases with little or no invasive tests and at a low cost. Nevertheless, the prevalence of LOPD is in line with previous studies [6, 7, 9, 10, 12–16, 18–24].

## Conclusion

Our study highlights the existence of a hidden population of LOPD patients in internal medicine departments who might benefit from early diagnosis and concurrent early initiation of potential treatments. Further national studies, involving larger cohorts of patients and centers, are required to corroborate our results of LOPD prevalence in Internal Medicine departments.

### Supplementary Information

**Additional file 1: Table S1.** Distribution of included patients by hospital and Autonomous Community in Spain. **Fig. S1** Distribution of included patients by Autonomous Community in Spain.

## Data Availability

Dataset generated and/or analyzed during the study are the property of the Sociedad Española de Medicina Interna. Anonymized datasets and related documents such as statistical analysis plan, protocol, and amendments can be shared upon reasonable request through a data sharing agreement. All requests should be addressed directly to Dr. Josep M. Grau-Junyent (JMGRAU@clinic.cat).

## Abbreviations

BMI: Body mass index
CK: Creatine kinase
DBS: Dried blood spot
GAA: Acid-alpha-1,4-glucosidase
IOPD: Infantile-onset PD
IQR: Interquartile range
LOPD: Late-onset PD
OSAS: Obstructive sleep apnea syndrome
PD: Pompe disease
SD: Standard deviation
ULN: Upper limit of normal
