# Supplementary material for: Screening for late-onset Pompe disease in Internal Medicine departments in Spain
Source: Orphanet J Rare Dis. 2023 Aug 31;18:256. doi: 10.1186/s13023-023-02887-z (PMC10472593; doi:10.1186/s13023-023-02887-z)
Supplement: Supplementary file 1 — Additional file 1: Table S1. Distribution of included patients by hospital and Autonomous Community in Spain. Fig. S1 Distribution of included patients by Autonomous Community in Spain. [file 13023_2023_2887_MOESM1_ESM.docx]

**Supplementary Table 1**. Distribution of included patients by hospital and Autonomous Community in Spain

| **Hospital** | **City** | **Autonomous Community** | **Number of included patients** |
| --- | --- | --- | --- |
| Hospital Clínic ^*^ | Barcelona | Catalonia | 80 |
| Lozano Blesa University Hospital ^*^ | Zaragoza | Aragon | 51 |
| Ramón y Cajal University Hospital | Madrid | Madrid | 40 |
| University Hospital Complex of Vigo | Vigo | Galicia | 30 |
| Málaga Regional University Hospital | Málaga | Andalusia | 29 |
| Dr. Peset University Hospital | Valencia | Valencian Community | 23 |
| Lucus Augusti University Hospital | Lugo | Galicia | 22 |
| Vall d'Hebron General Hospital | Barcelona | Catalonia | 11 |
| Puerta del Mar University Hospital | Cádiz | Andalusia | 10 |
| Puerta de Hierro-Majadahonda University Hospital | Madrid | Madrid | 9 |
| Araba University Hospital | Álava | Basque country | 8 |
| Vega Baja Hospital | Alicante | Valencian Community | 6 |
| León University Hospital | León | Castile-Leon | 3 |

^*^ Hospital where one patient with Pompe disease was identified

**Supplementary Figure 1**. Distribution of included patients by Autonomous Community in Spain


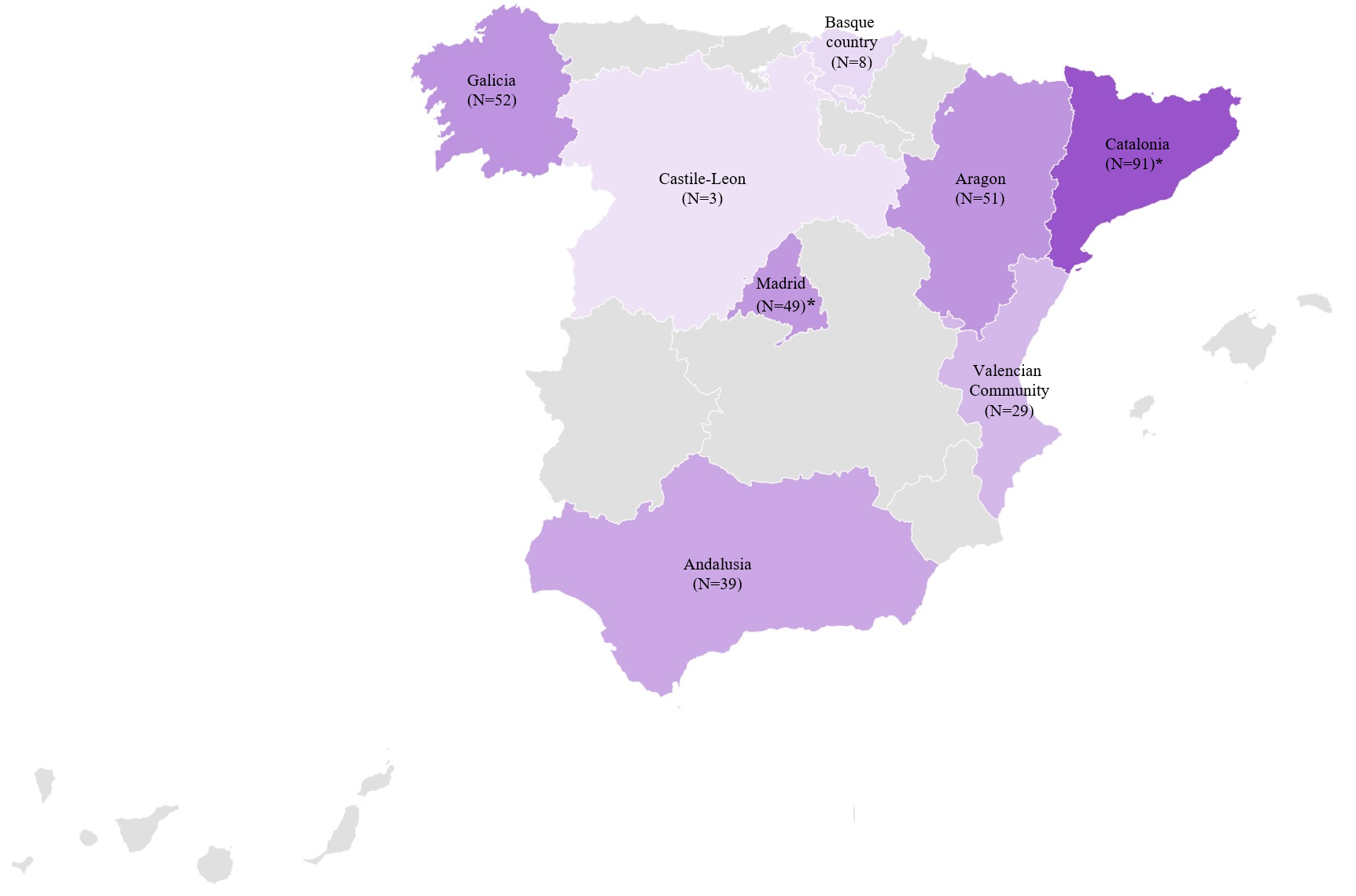


^*^ Hospital where one patient with Pompe disease was identified
